# Book Reviews

**Published:** 1803-02-01

**Authors:** 


### CRITICAL ANALYSIS

### OF THE

### RECENT PUBLICATIONS

### ON THE

### DIFFERENT BRANCHES OF PHYSIC, SURGERY, AND MEDICAL PHILOSOPHY.

---

### *Mr. Bell's Surgery, continued.*

An accurate knowledge of the arterial system forms so important a branch of the education of a surgeon, that our author dwells upon it with considerable, but not tedious minuteness; and previous to the consideration of aneurism, he lays before the reader, in the sixth Discourse, a general sketch of the arterial system and of the numerous inosculations whereby the circulating vessels are made to communicate through distant parts of the body.

The system of inosculation is one of the numerous beautiful contrivances of Nature for providing a remedy against some of the most dangerous injuries to which the human body is exposed; namely, interruption of the circulation through the great arteries, whether by disease or external violence. In all these cases it is well known, that the blood going to any of the extremities, if obstructed in its usual channel, finds its way through the minute inosculating branches given off from the larger artery above the point of obstruction, and thus supports the circulation necessary to the life of the parts, at first languidly, till these inosculating branches gradually enlarge, and from being secondary and subordinate ramifications, assume the office of principal branches, and at last become fully adequate to the purposes of life, growth, and perfect nutrition:

This important fact in the animal economy is now fully understood, and the valuable conclusions in the practice of Surgery to be derived from it, particularly with regard to the comparative safety of tying the larger arterial trunks, are equally familiar to the teacher and the practitioner. Mr. B. here describes anatomically the individual inosculations of the several branches of the circulating system, and lays particular stress upon three points in the arterial distribution which more peculiarly illustrate the doctrine of inosculation; these are the following.

1st. The communication between the axillary and the femoral arteries through the internal mammary, a branch of the former, which inosculates with the epigastric, a ramification of the latter. This arterial union between parts so widely distant as the upper and lower extremities is of peculiar interest, as it has been known to supply, in some degree, the want of an aorta, as is proved by a case cited by the author, of Mr. Paris, of the Hotel Dieu, who found in a subject which he injected, a remarkable contraction of the aorta a little below the arch, a consequent unusual enlargement of the arteries on the fore part of the chest, and a complete inosculation of the dilated mammary with the equally enlarged epigastric arteries. The whole of this dissection, which is contained in Dessault's Journal de Chirurgie, is highly curious and important.

2dly. The collateral branches belonging to the arteries of each extremity separately considered, and which are sufficient to keep up the circulation in the limb when the principal trunk is obstructed. Thus, the subclavian artery, before entering the axilla, sends off the thyroid and cervical arteries, which ramify round the joint, and mix their extreme branches with those which come off from the brachial artery, as may be shewn by tying the axillary artery and injecting the subclavian from within the thorax, when the wax will be found to run freely into the brachial artery below the ligature in the axilla. In like manner, in the lower extremity, the ischiadic, thyroid, and pudic arteries inosculate with ramifications from the profunda, and will supply the circulation when the external iliac artery is obstructed.

3dly. The lower joints of each extremity, the elbow and knee, are surrounded with their particular inosculations, which, though naturally more minute than those of the upper joints, so as to be shewn with difficulty by injections, are nevertheless capable of such rapid enlargement after obliteration of the larger arteries, as to supply the lower parts of the limb with perfect ease; as has been proved in numerous instances of operation for aneurism and other surgical cases, where it has been requisite to obstruct the larger arterial trunks.

Many desultory observations, and a profusion of cases, are added by the author, in illustration of the general principle of Inosculation; a principle now so thoroughly understood as to have introduced a much greater freedom in surgical operations in the tying of large arteries, and a full conviction of its general safety, as far as the future nutrition of the limb is concerned. Much has been done in a physiological point of view by Mr. Hunter's operation for aneurism, in explaining the vast resources for the nutriment of limbs which Nature possesses, in the power of rapidly enlarging inosculating branches and converting them into principal channels for the blood when the main trunks have been obliterated. The importance of this doctrine is very amply enforced by the author in this part of the work, and it immediately precedes the chapter on aneurism with very great propriety, as many circumstances relating to this most interesting disease are closely connected with and explained by the system of arterial inosculation. Several drawings of the arterial branches *in situ*, and of their mutual connexion are added, to assist the reader in the anatomical explanation.

The seventh Discourse begins the subject of Aneurism with a description description of the history and causes of this disorder. The spontaneous aneurism arising from a diseased and ossified state of the arteries come the first under consideration, and the example is given of the slow, gradual progress of aneurismal tumour in the ham, at first so small as to give no alarm to the patient, but by degrees increasing with all its formidable and characteristic symptoms; and when arrived at that point when the operation can no longer be delayed, suddenly succeeded by fresh aneurism in other parts of the arterial system, and finally proving fatal, without affording a chance of a permanent cure by any of the resources of art. Not unfrequently dissection shews an actual and manifest disease of the arterial system, the whole of which is thickened and brittle, and its cellular substance loose and spongy, with spots of ossification besetting the great arterial trunks, giving them the appearance of the knobbed crooked branches of an exhausted tree. Of this a very illustrative drawing is given by the author.

This predisposition however, he observes, seldom degenerates into aneurism without some degree of direct violence; and on this circumstance the author lays considerable stress, as it serves to explain the cause of the morbid appearances which attend the complaint. In all parts of the body there are weaknesses and imperfections which we are made sensible of only by the parts being hurt. Aneurisms of the limbs are never known in women; their system is lax, and they are generally exempted from those exertions by which the arteries are often injured or burst. Aneurism is rare in young men, and frequent in the middle stage of life, when the arteries become harder, when the muscular strength is unabated, and the occasional exertions of the limbs very violent. Aneurism is almost peculiar to men in the lower ranks of life, to soldiers, porters, labourers, miners, and those who work at laborious trades; and there are few of those enlargements of the artery which cannot be distinctly traced to some external injury, some blow, sprain, fall, or violent exertion of the limb. Hence the author infers, that even where no very decided disease of the arteries is present, a violent muscular exertion, unconnected with external wound, may burst the muscular coat of the artery and produce aneurism, the growth of which (confined as it is by the strong cellular sheath which encloses and strengthens the vessel) is often so slow and gradual as to have the appearance of spontaneous disease. Several cases are given in illustration of this particular point, namely, the slow growth of aneurism after an actual rupture of the artery; and the practical inference which the author deduces, is the importance of attending to the consequences of obscure sprains and internal injuries of the limbs, particularly the lower extremity, when succeeded by deep seated tumours, before the muscles, bone, and adjacent cellular substance are brought to that dreadfully diseased state which attends extensive extravasation from a ruptured artery.

A very interesting case, and several highly important observations follow, relating to the complication of caries of the bone, combined with, and depending on, aneurism of the contiguous artery. The case is communicated by Dr. Jeffry, anatomical professor at Glasgow:—A woman was ridden over in the streets, her arm broke in two places, and the bone much shattered. It remained absolutely neglected for six weeks, at which time it was examined, and found to be much swelled by a tumour reaching from the top of the shoulder to the arm, and pulsating in its upper part. It was opened in this spot, and pure blood flowed out; the wound however healed up, but the patient, after being apparently much neglected for five months, died. On dissection, which was eight months after the first accident, the arm was filled with a profusion of mixed and putrid blood, only two inches of the lower part of the bone retained its natural form, all the middle part of the bone was destroyed, and the head only remained on the upper part of the tumour, but with its cancelli quite eroded, nothing being left but the mere shell. Through the whole length of the bone the cancelli were dissolved, and many pieces of the bone were found in the heart of the tumour, exhibiting a most perfect specimen of caries produced by the presence of the extravasated blood which prevented the union of the callus; however, in the midst of this destruction, the periosteum had retained its life, and had made vast efforts at ossification, so as to form, in the length of the tumour, a large flat bone (not a remnant of the humerus, but a fresh production) of a fourth of an inch in thickness, three inches and a half broad, and six inches long, and in appearance not unlike the parietal bone of the skull. Beside this larger piece, the whole sac was full of specks of ossification within the centre of the membranes. The inosculating arteries had also performed their office, and had enlarged to a sufficient size to maintain the circulation.

At the conclusion of this discourse the author ingeniously infers, that, as aneurism more frequently arises from violence than disease, the lower extremity is more liable to this complaint than the upper, on account of its infinitely superior muscular force, whilst the artery of the thigh is long, tortuous, and not possessed of any power of resisting injury proportioned to the muscular exertions of the limb. The artery of the ham too is particularly endangered by the manner in which it perforates the tendon of the triceps.

A most important question occurs on the subject of aneurism, namely, whether amputation of the limb affords the patient a considerable chance for life. The ill success of this dreadful remedy has long made surgeons hesitate on its eligibility, even before the modern operation of tying the artery high up in its course was invented by Mr. Hunter. The authority of Guattani (which Mr. B. cites with partial prolixity) is strongly against amputation, and our author supposes an *inflamed* state of an aneurismal limb to exist, and to be an objection to amputation, from the great risk of gangrene; in the same manner as the inflammation succeeding to violent external injury also forbids amputation. In the latter case the inflamed state is produced by the extensive injury inflicted by extraneous violence; in the former (the author alledges with considerable force) a similar state is occasioned by the generally increased creased action of the inosculating arteries, on which the whole force of the circulation is thrown when the principal trunk is ruptured, and obstructed by the pressure of extensive extravasation, and in both the danger of gangrene from *active* inflammation is most imminent.

The method of operating in aneurism proposed by Mr. Hunter, and now in actual practice, but with some considerable variations, next engages our author's attention. The most important point in this operation is to determine whether the artery should be merely tied, or whether it should be cut through after the ligatures have been applied. The former was the practice of Hunter, and most of the surgeons who immediately imitated him; but the frequency of secondary hæmorrhages, from ulceration beneath the ligature, and the difficulty of obliterating the artery by simple ligature, rendered several of the most promising operations finally unsuccessful. On the other hand, when the artery is cut through, the state of the parts resembles more closely that of common amputation, in which the safety and practicability of securing even the femoral artery by simple ligature is fully proved. Hence it is that there appears good reason for adopting the method of dividing the artery before the wound is brought together; and the experience of Mr. Abernethy, and several eminent modern operators, appears in this respect perfectly to coincide with that of our author. We cannot however conclude this subject without directing the reader's attention to one circumstance consequent upon the division of the artery, which is, the danger of the ligature slipping off the divided end of the vessel, through the powerful retraction which speedily takes place, and which involves the hazard of almost instant death from the copious hæmorrhage through the newly opened vessel. For this there appear to be two remedies; the one, to include within the ligature a certain portion of cellular membrane along with the artery; the other, the simple, but apparently effectual, contrivance proposed by Mr. Cline, jun. of transfixing the artery with a needle, and passing by its assistance through the substance of the vessel, the same ligature which has already stopped the flow of blood by its compression. (*See the Medical Journal, No.* 41.)

In the last Discourses on the subject of Aneurism, the author treats of wounds of the arteries, and of the aneurism which forms over the wounded artery, along with general rules for the operations on great aneurisms and various anomalous cases. We shall not follow him through the whole of these pages, in which many valuable practical observations are given with freedom and spirit; but involved, as usual, in a profusion of words, perplexed by repetitions and want of method, and loaded almost to incumbrance with cases and quotations from writers of different ages.

Among the anomalous species of aneurism is that *by anastomosis*, as the author terms it, or an aneurismal tumour formed and fed by, not a single artery, but a great variety of vessels, all of which communicate freely with each other, and also pour their contents into into the tumour, and give it a most vigorous pulsation. If one of these tumours is opened, the hæmorrhage is prodigious, and the number of vessels which supply it is so great as to render all attempts at securing them individually by ligature, abortive. A curious case of this species of aneurism here related, is that of a young woman who has a tumour within the pelvis, lying between the rectum and vagina, principally inclining towards the left side, so that on introducing one finger within the rectum, and the other within the vagina, the pulsation may be felt between them. It has subsisted for some years, during which she has had two children. The lower hæmorrhoidal, and the pudic arteries, appear to have the greatest share in producing this aneurismal tumour, and by their increased activity the inosculations between them are so much enlarged, that pretty large arteries may be felt running a tortuous course along the walls of the rectum and vagina.

The cure of this species of aneurism, when seated on the forehead, or in accessible parts, is not, as our author observes, "to cut into it, but to cut it out." As it is a mere congeries of active vessels, small in dimensions, and almost innumerable in quantity, the interruption of particular vessels will not reduce the tumour; and, above all things, this variety of disease should be treated in its earliest stage, when alone it easily admits of radical cure.

The last part of this volume is occupied by the interesting subject of Fractures of Limbs, the analysis of which, with that of the expected second volume of this important work, we shall defer to a future opportunity.

---

*A Collection of Papers intended to promote an Institution for the Cure and Prevention of Infectious Fevers in Newcastle, and other populous Towns, &c. &c. by* JOHN CLARK, M. D. *Part the Second.* Newcastle upon Tyne.

IN a former number of our Journal, we briefly noticed the first part of this collection of papers, most of which were controversial between the medical gentlemen of the large and important town of Newcastle. The question in agitation was a local circumstance relating to fever wards, which involved the important consideration of the distance to which the contagion of infectious diseases in general, and of typhus fever in particular, could extend in ordinary cases, so as to be actively detrimental; a question of infinite moment wherever this disease is to be checked in its career by the assistance of medical skill, or prevented by civil regulations from extending its ravages.

The editor of this collection of papers adopts the opinion of the comparative security from contagion, which common precautions will ensure, and of the very limited extent to which the contagious atmosphere of the sick can extend; and in this second part numerous testimonials of the truth of this doctrine are given from authorities which have generally been deemed to carry the greatest weight. It is not necessary, however, again to direct the attention of of our readers to these, but rather to the concluding part of this collection, containing summary remarks on the means of exterminating typhus fevers and other contagious distempers, from towns, villages, boarding schools, &c. together with a supplement, containing numerous hints and remarks on the management and internal regulation of hospitals.

These pages, though few in number, are replete with good sense, and exhibit in their author a liberal humanity and public spirit, which must entitle him to the esteem and respect of all his fellow citizens; and of those in particular, whose names fill up the honourable list of the supporters of the benevolent institution, the improvement of which appears to be so warmly agitated.

---

*A Letter to Sir* WALTER FARQUHAR, *Bart. on the Subject of a particular Affection of the Bowels, very frequent and fatal in the East Indies.* 1801. 8vo. pp. 33.

SOME of the diseases that give the greatest alarm, by the direful rapidity with which they hasten to a fatal termination, and perplex the most by the indiscriminate urgency of opposite indications, are those which are produced in the constitutions of the inhabitants of cold and temperate regions, when removed to the burning influence of a tropical sun. Of this we have unfortunately had too many examples before our eyes; and we have still much, if not all to learn, respecting the best methods of guarding against these species of hazards to human life, necessarily incurred to a considerable extent, by our intimate connection with our East and West India Colonies.

An accurate observation of disease, and a fair trial of the various remedial powers, unbiassed by preconceived theory or partial judgment, are the contributions which we look for from our medical brethren, who devote themselves to the dangerous service of the tropical climates. Nor have they, at any time, been remiss in their endeavours to improve the knowledge of their art; and we cannot but regard the short description before us, as a very important and interesting communication, rich in matter of fact, and deserving of considerable attention.

The author, Mr. Fra. Duncan, relates the history of a very violent, and, hitherto, he conceives, unobserved inflammation in the bowels, attended with peculiar symptoms. The colon appears to be the part primarily and principally affected, and the inflammation is of the most active kind. The first symptoms are a severe fixed pain above the pubis, with extreme difficulty of making water, and frequently entire suppression of urine. The fever is very high, with unquenchable thirst and perpetual watchfulness; the pulse hard, frequent, and strong, like that of acute rheumatism, and the heat of the skin intolerably burning. The alvine discharge is considered by the author as affording a peculiar diagnostic symptom: There is from the first a violent and constant evacuation of a liquid

 matter, matter, which he compares to *water, in which raw flesh has been washed or macerated.* This evacuation, the seat of the pain, which is fixed above the pubis, and the difficulty of passing urine, the author regards as the three peculiarly characteristic symptoms; and in the severest form of the disease, the pain and evacuation from the bowels appear to come on at the same time. The progress of the complaint is rapid and violent, tenesmus generally attends the latter stages, but delirium scarcely ever, till the very approach of death. The tongue is at first white and furred, afterwards perfectly black. The other concomitant symptoms are described with great appearance of precision and accurate observation. The period of a total termination varies according to the circumstances of the disease and the degree of attention which can be given to the patient. In the fatigue and hurry of a camp in active service, death often occurs on the fourth, fifth, or sixth day, and never later than the twenty-first. It is not contagious, but sometimes epidemic. It attacks the young, robust, and those of a florid appearance and sanguine habit. In the late campaign against Tippoo Sultaun, this disorder carried off some of the finest young men in the army.

On dissection, the portion of colon directly beneath the seat of pain, was always found thickened, eroded, and ulcerated, and where emphysema came on before death, the bowel was found either wholly or partially mortified. In some instances, the marks of inflammation were found to extend to the small intestines, mesentery, and peritonæum: the liver was sometimes affected, and portions of white matter, of the size of a bean, dispersed through its substance: The bladder was never diseased, notwithstanding the severity of the dysuria or even strangury during the complaint. The omentum generally shewed marks of severe disease, and was often found dissolved into a black and putrid mass.

The author distinguishes this complaint from dysentery and from enteritis, by several very important diagnostic signs, and, we think, fully establishes the peculiar nature of the disease which he is describing.

With regard to the treatment, the author, guided by the analogy of most of the other bowel complaints of hot climates, began by giving purgatives; but he uniformly found that *they did irreparable mischief*, by increasing the inflammation, exhausting the strength, and thus hastening a fatal termination without at any time mitigating the violent symptoms. Disappointed in this remedy, the author, with great judgment, had recourse to the frequent use of emollient and anodyne glysters, together with constant warm fomentations of the belly, and mild diluent drinks. Blisters relieved the pain for a time, but it generally shifted to some other part of the colon. Blood-letting was of essential service in moderating the first inflammatory symptoms, but required great judgment in its use.

After the fever and pain were moderated, opium was of most essential essential service; as much so as it proved eminently mischievous in the first stages, whilst the pulse was hard and full, and the skin hot and dry; and this striking fact adds another most important example to the many instances, that shew the necessity of extreme caution in the use of opium in severe bowel complaints.

During the course of several fluxes in India, the colon is likewise subject to an inflammation, which the author terms secondary or symptomatic; and he takes some pains to distinguish it from the primary idiopathic disease which he has just been describing.

We have only to remark concerning this clear, simple, and highly valuable medical tract; that, in describing the peculiar pain of the abdomen, the author does not mention whether it is increased on pressure; and we could have wished a few more particulars concerning the appearances of the alvine discharge, especially whether it was attended with any peculiar smell or other marks, which have usually been ascribed to a state of acrimony or putridity.

The author concludes with great propriety: "To those young men who are preparing to go out to India in the medical profession, a knowledge of the facts here stated may be of some importance, and to them it cannot be deemed unseasonable in me to offer, what in their circumstances I should have gladly and thankfully received."

---

MEDICINA NAUTICA: *An Essay on the Diseases of Seamen; comprehending the History of Health in the Channel, for the years* 1799, 1800, *and* 1801; vol. iii. 8vo. pp. 507; *by* THOMAS TROTTER, M. D. *late Physician to the Fleet, &c.*

EVERY friend of his country, every one who forms a just estimate of the services of our brave tars, and of the importance of their exertions who guard the health of our fleets, must rejoice to see so able an advocate as Dr. Trotter continuing his labours to sooth their sick bed, and carry consolation into their retirement from the public service. Our Readers, we think, would have much cause to complain, if we passed over this interesting Work in our usual concise manner; we propose, therefore, to present them, at this time as well as occasionally hereafter, with a few extracts. The importance of the observations, which respect the health of seamen, will be collected from the manner in which, and the place where, they were made.

"The brave man," says Dr. T. "who exposes his life in the service of his country, has claims on the generosity of the public beyond every other competitor. Besides the dangers of battle, he has to contend with hardships and privations of all descriptions; and his diseases are of that class the most fatal in their issue. If therefore the language of truth and independence becomes any official situation, it belongs to the physician of a fleet or army. Of all conditions in human life, next to infancy, the sick bed is the most helpless. The finest examples of virtue have been practised there, as being the fittest field for the exercise of the most disinterested benevolence. To the medical profession, as connected with public public service, this office is almost exclusively confined. While this evinces the necessity of correct and punctual attendance, it also shows how minute and perfect all arrangements connected with it ought to be. But it appears in this work, even in a political view, that the subject loses nothing of its importance. And if confidence is to be bestowed on the officers of health, their representations ought to meet with nice attention and redress. I have thus been zealous to record many of my applications for correcting abuses; for it may be a long time before any succeeding physician of a fleet can have the experience which has fallen to my share; and if they are not inserted here, the hint for improvement may be lost. God forbid that I should carry to the grave a single idea that could benefit the naval service of the country! From that service I am now to retire, where I have spent all the best of my days, and to which my studies have been faithfully devoted. It must now think of me as a man who can have no share in its future operations, but who will be proud of its remembrance as long as he lives."

After a striking contrast between the labours of the political historian and the task which devolves on the historian of health, Dr. T. proceeds thus: "Amidst those scenes of war, desolation, disease, and death, it becomes the task of the medical philosopher and historian to collect such occurrences as point out the best means of enlarging the sphere of prevention, and by selecting the most prominent and striking facts, to establish rules that may direct future travellers and enquirers in the same path. Such has been the avowed intention of this work; in the execution of which we have freely thought for ourselves on every subject under review, where we wish our labours to be measured only by the spirit of utility which we inculcate. This being the case, the fastidious reader, if he wishes to follow us, must lay aside all technical formalities, and that species of grimace that staggers to unmask official iniquity. Having thus regenerated himself, and no longer the slave of prejudice, he will be able to discern modern improvements from ancient errors, and will be convinced, that the practice of medicine may really confer a great deal of benefit on mankind, without prescribing a single dose of physic. I will then conduct him through the horrors of the impress service; the orlop prison of the Liverpool guardship, and the press-room of a Bristol tender; through the Royal William and Cambridge receiving ships, that have so often sent abroad the vapours of contagion. I shall then lead him through the spacious, clean, dry, and airy decks of a ship of the line; where disease is prevented by the perfect obedience of every precept that commands health, where disorder is subdued by masterly discipline, and where the frown of an intelligent and accomplished officer can do more in the correction of crimes and immoralities, than a cat-o'-nine-tails. He shall afterwards view the pride of Nautical Medicine, the Markham Sick-Berth; where a diet is provided, delicate, restorative, abundant, at the seamen's seaman's own expence, that far exceeds the slow-paced improvements of an hospital ward. He will remark, in our walks through hospitals, that we have not hesitated to examine corners of darkness where the eye of observation never peeped before; the kitchen, the pantry, the pent up bed-house, the clothes of infection unventilated and unwashed, have all been dragged into day, with a thousand other imperfections that marked those neglected institutions. But our scrutiny and corrections have not been limited to ships and hospitals. He will observe, that we have even claimed protection for the heedless seaman, in the hours of his recreation and pleasures on shore, against the sink of vice and drunkenness, to which he was exposed by a wicked police; and two hundred gin-shops have been shut in Plymouth Dock, that have called down the execration of every thinking being on a magistracy, that dared to profane the functions of its office, in the very moment when sedition was lying in wait to corrupt the defenders of the country.

"If in making these digressions from the usual track of professional inquiry, that others have either not observed, or disdained to explore, much human affliction has been prevented or relieved; the head of the brave seaman laid on a softer pillow, or a new path to improvement opened, a candid reader will overlook a casual ruffled feeling or angry expression, during a struggle with all the powers and prepossessions which stupidity, obstinacy, and malevolence have opposed against us.* At the same time, let the reader separate the act of the executive officer of health in a fleet from that of the private physician, and distinguish between the duty of a public censor, that ought to wink at no abuses, and the forbearance that may be expected from an individual. My future retirement will then be secure from persecution and misapprehension; while my declining years shall be solaced with the thought, and look back with pleasure on the laborious but virtuous task, of having contributed to the comfort and happiness of thousands."

[ To be continued. ]

---

* It is curious to observe the hostility which has been offered, from different quarters, to my measures of safety in the fleet. A lady of rank, who was in the custom of giving large dinners to small parties, when the scurvy, in 1795, had put in contribution all the lemons and oranges of the country, complained that none could be procured for *her company*. "It is a shame," said her ladyship, "that the nation's money should be expended in this way; Captain P. tells me that these things are not good for sailors; and, what is worse, this physician can persuade Lord Howe to any thing." Ladies of another description, namely, the *green* women in Portsmouth market, combined *not* to sell a cabbage, or any thing, to the physician's servant. "Your master," said they, "has spoiled our trade by sending all the *sallad* to Spithead." When the gin-shops were shut in Plymouth Dock, it was prophesied that I should be "*found murdered in the streets*."

MONTHLY

